# Gene Expression in Late-Life

**DOI:** 10.3389/fgene.2012.00156

**Published:** 2012-08-28

**Authors:** Joseph L. Graves

**Affiliations:** ^1^Joint School of Nanoscience and Nanoengineering, North Carolina A&T State University, University of North CarolinaGreensboro, NC, USA

Science proceeds from its mistakes, just as well as its successes. We have argued above that it is “perfectly reasonable for natural selection to produce late-life plateaus in life-history characters.” This is premised on the forces of natural selection having fallen to very low values. The reality of late-life mortality plateaus was a revelation for me, especially having been one of the earliest critics of their existence (see Nusbaum et al., 1993). At that time I argued that aging consisted of an ever growing variety of physiological dysfunctions, which were ever increasing in their severity, leading to the eventual death of all individuals in a population.

Yet we now have both the well-corroborated observation of life mortality plateaus, as well as a series of theoretical developments and experiments demonstrating that antagonistic pleiotropy and mutation accumulation can account for these plateaus (Mueller and Rose, 1996; Reynolds et al., 2007; Mueller et al., 2011). This has led to a revolutionary recognition that aging is better described as the “detuning” of adaptation. Thus while a variety of physiological systems may detune during aging, there may be enough age-independent adaptations which allow some individuals to survive this life phase. For those that do, late life is now characterized as the phase in which adaptation re-stabilizes (as explained in Mueller et al., 2011) and thus their physiological performance is capable of allowing an undetermined length of additional life.

However this recognition has led us to entirely new and undiscovered country, specifically how does adaptation re-stabilize during late-life? Our previous work has focused on a variety of physiological, cellular, and molecular mechanisms which detune during the aging phase (Rose, 1991; Graves, 1997). Much of this work was described before modern whole genomic approaches and computational methods. At that time, we proposed that there must be suites of genes with age-associated expression related to organismal fitness undergoing age-specific decline. Subsequent work supports our original suppositions, even if this work has been carried out in *Drosophila* stocks of compromised quality with regards to elucidating generalizable patterns of aging (e.g., inbred and mutant strains; Girardot et al., 2006; Zhan et al., 2007) For example, Zhan et al. (2007) utilized microarray experiments to study gene expression in a variety of tissues (muscle, accessory gland, brain, testes, Malpighian tubules, fat body, and gut) in the *Drosophila melanogaster* w^1118^ mutant strain. They found that approximately 4–9% of all genes had an age-specific profile and different levels of up- and down-regulated genes with age in various tissues (Table 1).

**Table 1:** **Up- and down-regulated genes with age in various tissues of *D. melanogaster***.

**Tissue**	**Up-regulated**	**Down-regulated**	**Total**
Accessory gland	477	635	1255
Brain	380	452	832
Testes	429	394	823
Malpighian tubules	387	432	819
Fat body	339	323	662
Muscle	613	612	1255
Gut	305	282	587

This study also elucidated a number of genes that were age-associated and shared between different tissue types. An examination of the numbers of age-associated genes in this study suggests that many genes show age-independent expression profiles. For example, data from FlyAtlas suggests that about half the fly genome is expressed in all tissue types (Chintapalli et al., 2007). If this is so, then with an estimated *Drosophila* genome size of 14,000, we expect about 7,000 genes to be operational in all tissues. Indeed, Cherbas et al. (2011) examined the transcriptional diversity of 25 *D. melanogaster* cell lines. They probed 14,807 genes and found that 64% were expressed at a detectable level in at least one cell line. On average 5885 genes were detected (range 5398–6221). If we can rely on the Zhan et al. (2007) and Cherbas et al. (2011) studies to provide ball-park age-associated and tissue-specific gene expression profiles, then we can conclude that a very high fraction of (>75%) *Drosophila’s* genes show age-independent expression. Clearly there are methodological issues which will need to be addressed to determine more exact figures of age-associated gene expression in particular stocks living in specific environmental conditions. For example, it is also known that gene expression profiles differ between *Drosophila* males and females (Muller et al., 2011) and that evolutionary histories impact these profiles as well (Hutter et al., 2008). However, with all these sophistication aside, the existence of a genomic basis for a plateau in late-life survivorship is not too surprising. Clearly not all gene expression must shut down at later age, and if enough remains to sustain crucial gene-network function under sufficiently benign conditions, survival could go on for quite a long time.

These calculations suggest an immediate set of studies related to the genomics of the aging and late-life phases. It would be useful to use microarray studies to examine exactly how flies living in sufficiently benign environments transition at the genomic level into late-life. It is my own suspicion that an important aspect of this transitioning will be found among the control of transposable genetic element (TGE) expression (e.g., Murray, 1990). This will be particularly important in helping to apply the results of late-life research in *Drosophila* to humans, since there are documented patterns of TGE replication with age impacting human disease (Biemont and Vieira, 2006; Collier and Largaespada, 2007; Lowe et al., 2007; Fontana, 2010; Pornrutsami and Mutirangura, 2010).
